# Antibody evidence of SARS-CoV-2 infection in healthcare workers in the Bronx

**DOI:** 10.1017/ice.2020.437

**Published:** 2020-08-26

**Authors:** Elana R. Sydney, Preeti Kishore, Isaac Laniado, Lisa M. Rucker, Komal Bajaj, Michael J. Zinaman

**Affiliations:** 1 Department of Medicine, Jacobi Medical Center, Bronx, New York; 2 Department of Obstetrics and Gynecology, Jacobi Medical Center, Bronx, New York

The first recorded case of coronavirus disease (COVID-19) in New York City was on March 1, 2020, and by May 5, there were 171,723 confirmed cases with 13,724 confirmed deaths.^1^ The Bronx had the highest rates of hospitalization and death related to COVID-19 compared to the other 4 boroughs in New York City.^2^

Jacobi Medical Center, one of 11 acute-care facilities in the NYC municipal hospital system, is a 457-bed level 1 trauma center located in the Bronx with 3,225 healthcare workers. We began testing symptomatic employees on March 16 using the nasopharyngeal polymerase chain reaction (PCR) severe acute respiratory coronavirus virus 2 (SARS-CoV-2) test. In the first 6 weeks, we tested 1,264 employees, of whom 302 tested positive (23.9%). Recent reports showed that 12.2% of NYC healthcare workers had a confirmed positive SARS-CoV-2 PCR result.^3^

Preliminary data reveal that 19.9% of NYC residents have antibodies to SARS-CoV-2 virus, and 27.6% of Bronx residents have antibodies. Given this high prevalence of antibodies in the Bronx, we predicted that our staff would have higher prevalence of antibodies, especially those staff working in areas of perceived risk, such as the emergency department and critical care areas.

Once Abbott Labs (Abbott Park, IL) received the emergency use authorization for antibody testing using the SARS-CoV-2 IgG test,^4^ we began a voluntarily testing all employees at our facility. This test has a reported sensitivity of 100% and specificity of 99.6% when performed 2 weeks after symptom onset.^5^ Individuals were offered the IgG test as long as they were asymptomatic and had not had COVID-19 symptoms during the prior 2 weeks.

A retrospective chart review was performed to answer the following questions:

1. Were there demographic differences among antibody-positive healthcare workers?
2. What departments had the highest number of positive antibody tests?
3. What is the prevalence of antibody positivity in symptomatic healthcare workers?
4. How many of the asymptomatic healthcare workers developed antibodies?
5. What is the prevalence of antibodies in those healthcare workers with self-reported positive and negative SARS-CoV-2 PCR tests?

Data were extracted from the electronic medical record and were deidentified for analysis. In total, 1,700 healthcare workers were tested for SARS-CoV-2 IgG antibody between April 28 and May 4, 2020. We analyzed the data by looking at those healthcare workers that had positive antibodies and stratified it based on department, presence or absence of symptoms, and previously reported positive PCR.

Of the 1,700 individuals tested, SARS-CoV-2 IgG antibodies were detected in 327 individuals (19%). Among them, 221 healthcare workers with positive antibodies (67.6%) were women. The mean age of those that tested positive was 44 years (range, 22–73). The percentage of African-American healthcare workers that tested positive for antibodies was 27.2%. This was statistically significant compared to other racial groups that tested positive (*P* < .05). The highest prevalence (26% presence of IgG) was detected in the emergency department and the behavioral health department (Table 1). The behavioral health department included the psychiatric emergency room and inpatient and outpatient psychiatry. Suprisingly, the lowest prevalence was found in critical care staff, with 11% testing positive.

**Table 1.** Results of Antibody Positivity by Department, Presence or Absence of Symptoms and Reported Negative PCR Test

Department	Total	Positive IgG (%)	Symptomatic		Asymptomatic		Symptomatic Negative PCR	
			Total	Positive IgG (%)	Total	Positive IgG (%)	Total	Positive IgG (%)
Ambulatory care	232	51(22)	143	38(27)	89	13(15)	143	16(11)
Behavioral health/Psychiatry	104	27(26)	65	22(34)	39	5(13)	65	16(25)
Critical care/ICU	418	47(11)	201	36(18)	217	11(5)	201	13(6)
Emergency department	309	80(26)	177	65(37)	132	15	177	32(18)
Medical-surgical unit/Inpatient	219	34(16)	122	30(25)	97	4(4)	122	6(5)
Not applicable	26	10(38)	12	6(50)	14	4(29)	12	3(25)
OB/Gyn	81	15(19)	38	12(32)	43	3(7)	38	3(8)
Other healthcare worker	296	61(21)	137	41(30)	159	20(13)	137	16(12)
Trauma service	15	2(13)	12	2(17)	3	0(0)	12	1(8)
Total	1700	327(19)	907	252(28)	793	75(9)	907	106 (12)

Note. PCR, polymerase chain reaction; ICU, intensive care unit; OB/Gyn, obstetrics and gynecology.

More than half (53%) of the individuals tested reported having symptoms suggestive of COVID-19. Of these healthcare workers, 28% showed the presence of IgG antibody, and 9% of asymptomatic healthcare workers had IgG antibodies. Notably, 12% of those who tested positive for the presence of IgG reported a negative SARS-CoV-2 PCR result. As expected, 92% of individuals that reported a positive PCR test developed IgG antibodies. A small number of individuals, representing 1% of those reporting a positive SARS-CoV-2 PCR test prior to being tested, had a negative antibody test. This finding is consistent with a previous report from China in which a small number of patients with COVID-19 did not develop antibodies.^6^

Our results reflect a higher overall rate of SARS-CoV-2 antibody development among healthcare workers in the Bronx compared to reported rates in NYC healthcare workers.^3^ However, the rates were lower than the reported Bronx community prevalence.^4^ This finding is not unexpected because hospital staff had better access to protective equipment than did the general population as well as a heightened awareness of the seriousness of the infection. Based on our findings, hospitals with psychiatric services, especially with psychiatric emergency departments, should consider increasing the use of SARS-CoV-2 transmission prevention resources. Our African-American healthcare workers had a significant difference in antibody positivity. This finding needs to be confirmed in a larger study, and additional investigation is necessary to understand the reasons for this finding. The low rate of antibody development in critical care areas could be explained by the controlled environment and lower volume compared to other areas. Finally, 12% of hospital staff who developed antibodies in spite of a negative PCR test could be explained by false-negative PCR testing, infection after the PCR test, or inaccurate self-report.

It is unclear whether the presence of IgG antibodies confers long-term immunity. Emphasis is being placed on antibody testing for reopening the economy and return-to-work policies.^7,8^ However, only 1 in 5 healthcare workers developed antibodies during the peak of the pandemic at our hospital; thus, the utility of antibody testing to guide staffing considerations is limited. Ultimately, development of prophylactic treatments and therapies for COVID-19 is needed to ensure the safety of our healthcare workers pending the arrival of a vaccine.
